# Observations on Eclampsia

**Published:** 1883-06-20

**Authors:** H. L. W. Burritt


### OBSERVATIONS ON ECLAMPSIA.

BY H. L. W. BURRITT, M. D.

In the Compendium for January, the question of "Acceleration of Puerperal Convulsions (page 73) is stated to be an open one. I take it for granted that by a large majority of the profession the following propositions are considered axioms :

1st. That bleeding *pro re nata*, as to quantity and according to constitution and strength, is indispensable, and its neglect almost criminal.

2d. That delivery the sooner the better is always the rule and not the exception, in all cases of eclampsia.

3d. That bleeding being premised, delivery will end the convulsions after half an hour.

4th. That delay is far more dangerous to the patient than the use of any means, manual or instrumental, to the end.

Hence I cannot see any force in the foreign authority or statements, "that delivery does not, as a rule, exert a favorable influence on puerperal convulsions," and that statistics, as given by Dr. F. Schauta, are unreliable, as his own exceptions—relief of pressure, restoration to the normal state, safety to the child, all deny his facts as given.

All experienced physicians have seen cases of convulsions in women seven to eight months pregnant, where even after bleeding the convulsions persisted until the os was forcibly dilated and the child turned and delivered, often with all the force of traction the attendant could use. I have seen thirteen of such cases with out any bad result to the mother. In one case there were fourteen convulsions, six after bleeding to eighteen ounces—seven and a half months—fourth child; weight, two pounds eight ounces—forcible dilatation—time, half an hour—only one spasm after loss of consciousness—child now five years old.

Turning is far preferable to the forceps, as there is no obstetrical instrument equal to, and no force so great, that can be used so safely, both in dilatation and extraction, as the *thinking* human hand; its power is sure, and it knows what it grasps, and it gives confidence to the operator. The experience of over a hundred cases justifies my opinion. Who ever heard of a case by a *regular* of injury to the mother where the hands were used alone? Is it not true that ergot carefully used, rupture of the bag of waters, and bleeding followed, by early and forcible extraction, have saved more mothers and children in eclampsia than any other method? Bleeding to save the brain, ergot as safety against the hemorrhage of chloroform, forcible extraction to relieve those terrible spasms, immediate relief where an hour's delay may be fatal, and bleeding, above all the anchor of safety—are not these *now* the established *rules* of the profession?—*Med. and Surg. Rep.*

